# Treatment of Goitre by Chloride of Ammonium

**Published:** 1880-05

**Authors:** 


### TREATMENT OF GOITRE BY CHLORIDE OF AMMONIUM.

Dr. Stevens, of Dunham, Canada, states that he has employed chloride of ammonium in the treatment of seven cases of common goitre, or simple hypertrophy of thyroid gland, with most surprising and satisfactory results. Six of the patients were girls under twenty years of age, and all of them were entirely cured after about three months of treatment. The seventh case was that of a married woman, aged forty, and the mother of several children. The tumor in this case was of enormous size, and the patient suffered a good deal from disturbances of respiration and circulation. She took the chloride two or three months, and at the end of that time the bronchocele was reduced one-fourth in size, and all the circulatory and respiratory symptoms were relieved. Treatment was discontinued, because she became pregnant. The dose used in all the cases was ten grains three times a-day, but Dr. Stevens thinks, larger doses might be useful in old cases. No other medicine or hygienic treatment was combined with the chloride of ammonia. In the cases of the six girls, the tumor had made its appearance about puberty, but in none of them was there any evidence of menstrual derangement or uterine disease.—*Canada Med. Record*, Feb. 1880.

